# Ultrasound Evaluation of Gastric Emptying and Comparison with Patient-Reported Risk Factors in Elective Surgical Patients

**DOI:** 10.3390/jcm14020641

**Published:** 2025-01-20

**Authors:** Sezgin Inan, Basar Erdivanli

**Affiliations:** Department of Anesthesiology and Reanimation, Faculty of Medicine, Recep Tayyip Erdogan University, 53100 Rize, Turkey; dr.sezgininan@gmail.com

**Keywords:** gastric emptying, stomach, ultrasonography

## Abstract

**Background/Objectives**: Despite standard preoperative fasting guidelines, residual gastric content can persist in some patients, increasing the risk of aspiration pneumonitis. Multiple patient-specific factors may predict gastric content retention, but their predictive accuracy is limited. We hypothesized that ultrasound would more reliably identify residual gastric content compared to a comprehensive questionnaire and aimed to determine the most practical approach for risk assessment in elective surgical patients. **Methods**: We conducted a prospective, observational study in adult patients scheduled for elective surgery at a single center. All participants adhered to an 8 h fasting period. The primary outcome was the incidence of a “full stomach” on ultrasound. Secondary outcomes included the Perlas risk classification, comparisons of established volume estimation formulas, correlations with self-reported fasting duration, agreement between questionnaire-based predictions and ultrasound findings, and the time efficiency of each method. Multivariable logistic regression and Cohen’s kappa were used for analyses. **Results**: Data from 404 patients were analyzed. Despite prolonged fasting, 16.3% had a full stomach by ultrasound, suggesting incomplete gastric emptying. Early satiety and cholelithiasis significantly predicted a full stomach; prolonged fasting duration and female sex were protective. Questionnaire-based predictions demonstrated fair agreement with ultrasound (kappa = 0.327). The Michiko formula often yielded negative volumes, highlighting limitations in volume estimations. Ultrasound examination (3 min median) was faster than questionnaire completion (5 min). **Conclusions**: Ultrasound accurately detects residual gastric content, outperforming questionnaire-based assessments. Integrating it into routine preoperative evaluation may improve patient safety, although research is needed to refine volume estimation formulas and expand feasibility for patients with positioning limitations.

## 1. Introduction

Ensuring gastric emptying by observing an adequate fasting period is a widely employed and cost-effective strategy to minimize the risk of complications such as pulmonary aspiration during anesthesia [1,2]. Recent European guidelines similarly stress the foundational role of preoperative fasting in preventing pulmonary aspiration but call for individualized strategies [3]. Several studies suggest that patients fast for longer periods than recommended [4,5]. However, even these prolonged fasting times do not always result in complete gastric emptying, and residual content may persist in some individuals despite adherence to fasting recommendations [6,7].

Multiple factors beyond fasting duration have been identified as influencing gastric emptying. These include medical conditions such as gastroparesis, as well as factors related to dietary intake, such as the consumption of high-fat meals [8,9]. Other factors, such as age, medications, pregnancy, and disorders affecting the dopaminergic system, can further contribute to delayed gastric emptying and the retention of residual gastric content [10,11,12,13]. The varied degree and insidious nature of pulmonary aspiration also makes it difficult to identify the risk factors. As an example, although gastric emptying was shown to be delayed in diabetic patients, there is not enough evidence to state that pulmonary aspiration risk is increased [14,15]. Similarly, studies suggest either delayed or non-delayed gastric emptying in obese patients, yet no additional risk of pulmonary aspiration of gastric content [16,17,18].

Traditionally, identifying these risk factors relies heavily on specific patient characteristics known to influence gastric emptying. Identifying patients at risk of retaining residual gastric content is critical, as the presence of such content can lead to serious complications if perioperative care is suboptimal [19]. The predictive power of these characteristics remains limited, as they do not always correlate with actual gastric content, particularly when relying on patient-reported data alone. Ultrasound (USG) has emerged as a non-invasive, real-time imaging tool capable of assessing gastric volume, offering a more objective measure of residual gastric content [20]. Over the years, formulas based on USG measurements have been developed to estimate gastric fluid volume, enhancing the utility of USG in preoperative assessments [21]. Despite reported strong correlations (r ≈ 0.82–0.86) between formula-derived estimates and actual suctioned gastric volumes in certain populations [22], concerns remain regarding the reliability of these formulas across different clinical contexts. In particular, the formulas may overestimate small residual volumes while underestimating large ones, potentially limiting their usefulness in risk stratification for aspiration. Consequently, while volume estimation formulas offer a non-invasive quantitative metric, their real-world performance warrants further investigation—especially among diverse surgical populations with varying body habitus and comorbidities.

We hypothesized that USG would be more reliable than a questionnaire in identifying patients at risk for residual gastric content. This study aimed to evaluate the correlation between patient-reported risk factors and USG findings to determine whether a questionnaire-based approach could serve as a reliable predictor of residual gastric content. Our goal was to assess the pragmatic reliability of USG, not only as a diagnostic tool for visualizing gastric content but also as a method for volume estimation, based on established and study-derived formulas. By comparing these methods, we aim to offer insights into the most effective, practical, and reliable approach for preoperative gastric assessment, with a particular emphasis on patient safety, ease of implementation, and time efficiency.

## 2. Materials and Methods

The study was registered in clinicaltrials.gov (trial registry number NCT06606782) and conducted at a tertiary hospital between 5 October 2020 and 10 October 2022. All adult patients (≥18 years) scheduled for elective surgery at the Recep Tayyip Erdoğan University Rize Training and Research Hospital were invited to participate. Patients who adhered to preoperative fasting guidelines (minimum 8 h of fasting) and provided written informed consent were included. Exclusion criteria included children under 18 years of age, pregnant women, patients requiring emergency surgery, patients with a history of previous stomach surgery, patients who did not complete the sufficient fasting period (less than 8 h), patients with whom reliable cooperation for questionnaire completion could not be established (e.g., inability to provide reliable answers), patients who could not be positioned on their right side due to clinical conditions, and patients in whom sufficient quality imaging of the gastric antrum could not be obtained.

The primary objective of this study is to determine whether USG effectively identifies residual gastric content in patients who have adhered to standard preoperative fasting guidelines. The primary outcome measure is the incidence of a “full stomach” based on real-time USG visualization (solid or fluid content). The secondary objectives are to investigate the feasibility and accuracy of multiple ultrasound-based volume formulas (Michiko, Bouvet, Perlas) by comparing their calculated volumes with both the USG findings and the Perlas risk classification (Grades 0, 1, 2). We also seek to evaluate whether a comprehensive patient-reported questionnaire offers predictive value for detecting residual gastric content and aligns with USG results. Finally, we will compare the time required to perform ultrasound assessment versus completing the questionnaire, thereby assessing the efficiency and practicality of each approach.

### 2.1. Literature Review, Questionnaire Development, and Testing

A literature review was conducted between 10 June and 31 August 2020 to identify factors associated with gastroparesis and delayed gastric emptying. These findings informed the creation of a comprehensive questionnaire to assess patient-related risk factors. Based on the literature review, the questionnaire was developed in consultation with the Department of Gastroenterology and consisted of 69 items under seven main headings, covering potential risk factors such as medical history, dietary habits, and gastrointestinal symptoms (Appendix A.1). One caveat was that autonomic dysfunction can present with diverse symptoms, some of which can develop gradually and are often underreported [23]. Using laboratory testing to confirm autonomic dysfunction is possible but time-consuming and not routinely performed [24]. Our pilot testing showed that including a comprehensive tool like the Composite Autonomic Symptom Scale alone required significantly longer screening time (≥30 min), conflicting with our goal of a feasible questionnaire. Given the focus on perioperative screening in a high-throughput working environment, we prioritized items most directly related to gastric emptying. Therefore, we excluded certain autonomic dysfunction-related factors such as erectile dysfunction, urinary retention/incontinence, and sicca (dry mouth, dry eyes), which are not relevant to our main outcome, to maintain a practical questionnaire length. The questionnaire was pilot tested with hospital personnel to assess its feasibility and ease of administration.

### 2.2. Ultrasonography Training

To ensure the reliability and consistency of USG measurements, the primary investigator received five days of dedicated training between 1 September and 7 September 2020 in the Radiology Department, focusing on gastric antrum anatomy, gastric ultrasound imaging, standard scanning windows (supine and right lateral decubitus position) and the accurate measurement of the cross-sectional area. After the training period, the primary investigator performed 20 supervised scans on volunteer patients (including a case presenting with a history of gastrectomy) between 10 September and 2 October 2020. The scans were verified by the same radiologist to achieve a concordance rate of ≥90%.

### 2.3. Ultrasound Measurements

Ultrasound measurements were performed to estimate the volume of residual gastric content by measuring the gastric antrum cross-sectional area in both the supine and right lateral decubitus positions during the gastric relaxation phase. Measurements included the serosal layer and followed the methods described by Bolondi and colleagues [25]. All measurements were performed at the relaxed state of the stomach. To ensure this, one peristaltic wave was observed before the measurements.

Three USG-based formulas were used to estimate gastric residual volume. Michiko’s formula was validated for healthy adults but is considered less suitable for diverse patient populations [26]. Bouvet’s formula involves a single measurement in semi-sitting position but was reported to have low correlation with actual gastric volume [21]. Perlas’ formula, validated for patients with a body mass index (BMI) below 40 kg/m^2^, was reported to yield more reliable results [27]. The formula derived by Kruisselbrink and Perlas was not used as it is validated for obese patients.

### 2.4. Questionnaire Administration and Blinding

After confirming participant eligibility, each patient was asked to complete the risk-factor questionnaire in the preoperative preparation room, under the supervision of an anesthesia technician, who was also responsible for obtaining other items like the American Society of Anesthesiologists physical score and scheduled operation. The ultrasound examination was performed in the operating room to enhance patient privacy, as it required exposing the epigastric region. The examination was performed by the principal investigator, who did not have access to the completed questionnaire. This workflow minimized potential bias by blinding the ultrasound operator to questionnaire-identified risk factors.

### 2.5. Determining the Risk for Aspiration of Gastric Content

The Perlas risk classification for aspiration risk was calculated by stratifying each participant as Perlas Grade 0, 1, or 2 based on gastric USG findings. This step included calculating estimated volume of gastric content using Perlas’ formula. Grade 0 indicates no visible content, Grade 1 denotes clear fluid in right lateral decubitus (RLD) position only, and Grade 2 signifies solid content or fluid visible in both supine and RLD positions, or fluid volume > 1.5 mL/kg [28].

### 2.6. Statistical Analysis

Sample size was calculated using the G-Power program (version 3.1.9.7) for the primary outcome: the incidence of a “full stomach” on preoperative gastric ultrasound. We assumed an approximate 15% incidence of full stomach based on the previous literature. We planned to include up to four key covariates in a multivariable logistic regression model; thus, we assumed that 40–60 full stomach events would be required to minimize overfitting. Therefore, our total sample size target was calculated to be between 266 and 400 participants. To account for possible dropouts or inadequate imaging, we aimed for at least 400–500 enrolled patients.

Data analysis was performed using the R statistical program (Version 4.1.2). Normality of numeric data was assessed using the Kolmogorov–Smirnov Test. Normally distributed data were presented as mean ± standard deviation, while non-normally distributed data were presented as median (interquartile range) or median [limits]. Categorical data were presented as numbers and percentages.

Patients were divided into two groups (empty vs. full stomach) based on ultrasound findings, and into three groups (Grade 0, Grade 1, Grade 2) based on Perlas’ qualitative risk scoring. Logistic regression models were used to identify significant predictors of gastric content and Perlas risk.

The relationship between gastric volume estimation formulas and aspiration risk was explored using logistic regression. Receiver operating curve (ROC) analysis was used to assess model performance, with area under the curve (AUC), sensitivity, and specificity reported. The best cutoff for each model was determined using Youden’s index. Patients were initially categorized into three groups—empty, fluid, or solid content—according to ultrasound visualization. Because only 9 patients presented with solid gastric content, we could not implement a three-category (empty/fluid/solid) logistic model. Instead, fluid and solid contents were combined into a single “full stomach” grouping for binary logistic analysis. For aspiration risk, Perlas Grades 0 and 1 were similarly combined into a single “low risk” grouping to enable a binary logistic analysis against Grade 2.

To evaluate the agreement between questionnaire-based predictions and USG findings, Cohen’s Kappa was calculated. The time efficiencies of both the questionnaire and USG were compared with descriptive statistics to report average times for each method. A *p*-value of <0.05 was considered statistically significant.

## 3. Results

**Figure 1:**
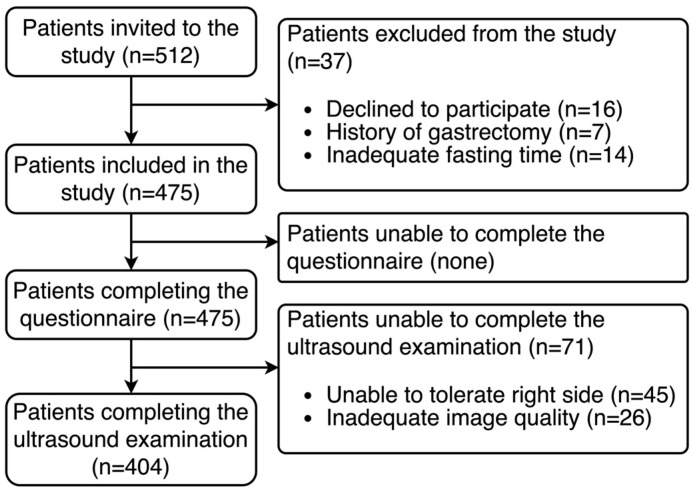
STROBE diagram illustrating participant recruitment, exclusions, and study completion.

Data from a total of 404 patients were analyzed. A STROBE diagram is given in Figure 1. Briefly, no patients were excluded due to events related to the questionnaire. However, 45 patients (9.5%) could not complete ultrasound examination due to not being able to tolerate right side position. The comparison of the demographic and clinical characteristics of these patients is given in Table 1. Briefly, there were no significant differences in risk factors, except that patients unable to tolerate the right side position had lower extremity fractures.

Patient characteristics are given in Table 2. Body mass index was slighly higher in patients with a non-empty stomach (*p* = 0.051). There were 65 patients (16%) who had a body mass index above 35. Sixteen of them (25%) had a non-empty stomach (*p* = 0.074) and only six of them (9%) had a moderate or high risk of aspiration (*p* = 0.566). It was noted that the fasting duration was longer than suggested by the guidelines. The mean duration of fasting was 6–90 min longer in patients with an empty stomach (*p* = 0.021). It was noted that time to surgery was significantly longer in patients who had a high risk of aspiration. In 13 patients, the time to surgery (preoperative preparation) was longer than 2 weeks. Five of them had cancer surgery and waited for additional imaging. The rest had to wait for optimization of medical therapy: five patients had hypertension, one patient had diabetes mellitus, one patient had coronary artery disease, and one patient had hyperthyroidism. There were no patients with a history of pancreatic or duodenal surgery.

**Figure 2:**
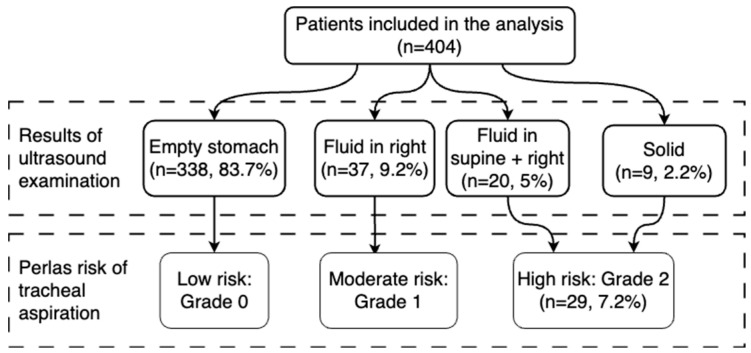
Flowchart summarizing the gastric ultrasound results among the 404 analyzed patients. Patients are categorized into “Empty stomach” vs. “Non-empty stomach”. For non-empty stomachs, ultrasound findings are subdivided into fluid or solid content, the body position(s) in which the fluid was visualized, and the corresponding Perlas risk grade (0 = Low, 1 = Moderate, 2 = High). The values in parentheses indicate the percentage of the total cohort (n = 404).

Figure 2 illustrates the distribution of ultrasound findings and corresponding aspiration risks. In total, three hundred thirty-eight patients (83.7%) were categorized as having an empty stomach (Perlas Grade 0, low risk), thirty-seven (9.2%) with fluid visible only in the right lateral decubitus position (Grade 1, moderate risk), twenty (5.0%) with fluid in both supine and right lateral positions (Grade 2, high risk), and nine (2.2%) with solid content (also Grade 2). Thus, sixty-six patients (16.3%) presented with a non-empty stomach, a higher proportion than initially expected given the adherence to 8 h fasting.

Ultrasound findings are given in Figure 2. Briefly, a full stomach was identified in 66 patients (16.3%) and the anesthesia team was notified. The risk of aspiration was identified as baseline in 355 patients (87.8%), low in 37 patients (9.2%), and high in 12 patients (3%).

The antrum area and estimated gastric volumes according to three different formulas were presented in Table 3. It was noted that Michiko’s formula yielded negative results. Bouvet’s formula yielded no negative results but the results for both aspiration risk categories were similar. Perlas’ formula yielded some negative results for patients with moderate risk of aspiration (Perlas risk score 1). The relationship between different volume estimation formulas and the Perlas risk score was presented in Table 4. Michiko’s formula was the only one with a positive relationship, suggesting higher estimated volume may be associated with a higher risk of aspiration and the result is significant (*p* = 0.040). However, the result is weak and the Akaike value is very high (358.98). Bouvet’s and Perlas’ formulas had lower Akaike values (both around 75), but their relationship was negative.

**Figure 3:**
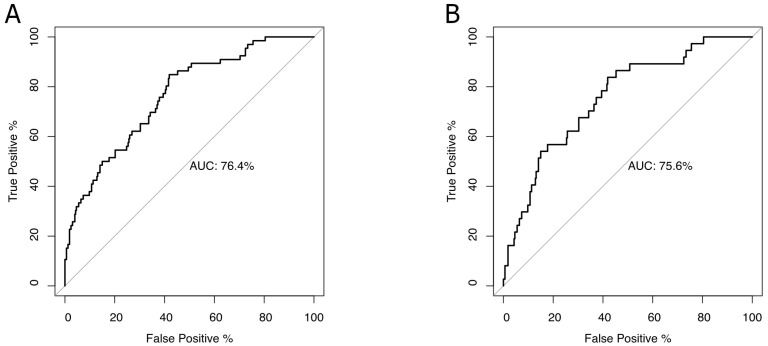
Receiver operating characteristic (ROC) curve for predicting a full stomach (Panel (**A**)) and Perlas risk (Panel (**B**)) ROC curves displaying the performance of the logistic regression models in predicting (**A**) a non-empty stomach (AUC 76.4%) and (**B**) a moderate-to-high Perlas risk (AUC 75.6%). The diagonal line indicates chance-level discrimination. Model thresholds were determined using Youden’s index to optimize sensitivity and specificity.

Significant patient-related predictors of a full stomach based on USG findings are given in Table 5. Early satiety and cholelithiasis had a significantly positive association. Fasting duration and female sex had a negative association. The model had an AUC value of 76.37. The best cutoff based on Youden’s index was 0.13, which yields a sensitivity of 84.8 and specificity of 58.1 (Figure 3A).

Significant patient-related predictors of Perlas risk score are given in Table 6. Early satiety and cholelithiasis had significantly positive association. Fasting duration and female sex had a negative association. The model had an AUC value of 75.55. The best cutoff based on Youden’s index was 0.13, which yields a sensitivity of 83.8 and specificity of 58.1 (Figure 3B).

### 3.1. Agreement Between Questionnaire Predictions and USG Findings

The agreement between the questionnaire-based predictions and USG outcomes was evaluated using Cohen’s Kappa on a data from 393 patients, yielding a Kappa value of 0.327, indicating fair agreement with a significant *p*-value (*p* < 0.001). This suggests that the questionnaire aligns with USG results better than chance and does provide some predictive value but is not sufficient on its own to replace USG.

### 3.2. Time Efficiency

The time required for the questionnaire and the USG examination was recorded for 404 patients. Forty-five patients who could not assume a right-side position and twenty-six patients whose images were of insufficient quality were excluded from the analysis. The median duration for the USG exam was 3 min (IQR, 2–4; range, 2–6). Notably, some elderly patients (>70 years) required up to 5 min to capture a full peristaltic wave, irrespective of body mass index or room temperature. In contrast, the questionnaire required a median of 5 min (IQR, 4–7; range, 4–15). The difference in completion times was statistically significant (*p* = 0.02).

Among younger patients (aged < 40 years, n = 82), both methods tended to be faster, with a median of 2 min for USG and 4 min for the questionnaire. In contrast, patients over 70 years old (n = 52) needed at least 4 min for USG and 6 min for the questionnaire. Participants who reported multiple medication use (n = 40) needed more than 10 min to complete the questionnaire, as each medication had to be verified in the patient’s chart or electronic health record.

## 4. Discussion

This study demonstrated that USG is an effective and accurate tool for assessing residual gastric content in patients adhering to preoperative fasting guidelines. However, 8.8% of participants with fractures or other mobility constraints could not be examined further. This is a major shortcoming of USG examination, as a recent meta-analysis with 1203 patients concluded that 10 cm^2^ measured in the RLD position can serve as an easy-to-obtain finding to suggest inadequate gastric emptying [29].

Many patients fasted longer than the recommended guidelines, yet 16.3% still showed ultrasonographic evidence of a full stomach, which is in line with the literature [30,31]. This finding underscores that simple adherence to fasting rules does not ensure complete gastric emptying. Prolonged fasting may reduce risk overall, but factors such as early satiety, or cholelithiasis can override the protective effect of extended fasting. Hence, strict adherence to fasting guidelines is foundational, but not fail-safe, and may be supplemented by ultrasound assessment. Logistic regression analysis revealed that early satiety was a significant predictor of a full stomach while fasting duration and female sex had a protective effect. The finding that early satiety was associated with a full stomach may indicate subtle gastric dysmotility or impaired gastric accommodation, as patients who feel prematurely full sometimes harbor delayed gastric emptying or partial gastroparesis [32].

Interestingly, diabetes mellitus, an important risk factor for gastric paralysis, was not found as a significant risk for stomach fullness. This finding is contrary to a recent observational study by Zhou et al. [33]. However, Zhou et al. compared patients with only 6 h fasting, which may have been inadequate for diabetic patients. Additionally, Zhou et al. suggested that diabetes-related eye disease—suggestive of autonomic neuropathy—may be more susceptible. However, our analysis showed no relation with any questionnaire item related to autonomic dysfunction.

A review by Goyal et al. highlights some of the mechanisms related to slow or rapid gastric emptying in diabetic patients [34]. Put simply, chronic hyperglycemia and oxidative stress may cause either slow or rapid gastric emptying, but it is acute hypoglycemia or hyperglycemia which may cause rapid or slow emptying, respectively. One logically expects no acute hypoglycemia but rather slowly developing hyperglycemia in preoperative patients with diabetes. Unfortunately, the questionnaire is not designed to capture this level of detail.

Qualitative scoring using the Perlas risk scale showed moderate to high aspiration risk in 12% of patients. The analysis of gastric volume estimation formulas highlighted limitations, with significant discrepancies between estimated volumes and aspiration risk, indicating a need for more accurate and reliable formulas in clinical practice. Specifically, the two most cited formulas (Bouvet and Perlas) had a negative relationship, meaning higher volume estimates are associated with a lower risk of aspiration. Many current ultrasound protocols estimate gastric volume primarily from antral cross-sectional measurements because the antrum is often accessible, relatively easy to visualize, and has been validated in certain cohorts [27,35]. Some researchers have proposed multi-view scanning or even 3D ultrasound approaches that visualize a larger portion of the stomach, suggesting that antrum-only formulas could overfit certain populations or misrepresent extremes of gastric volume [21,26]. Our analysis similarly indicates that relying strictly on antrum-based calculations may introduce inaccuracies. We are of the opinion that clinicians may benefit from the direct visual assessment of USG for identifying residual gastric content, rather than relying on volume estimates that may fail to reflect true aspiration risk in diverse patient populations.

Both our logistic models demonstrated moderate overall predictive accuracy, albeit with different strengths. Early satiety and cholelithiasis were positively associated with higher risk in each model, suggesting that specific patient-reported symptoms may warn the clinician to gastric retention. The protective effect of prolonged fasting duration aligns with conventional wisdom that lengthier fasting reduces content. The negative association with female sex in the Perlas model might reflect physiological differences in gastric motility or simply an artifact of our sample’s demographics, as the ratio of female participants in Perlas et al.’s study was larger compared to our study (two third vs. one third) [27]. Nevertheless, these associations underscore the potential value of integrating patient-reported factors (satiety patterns, biliary disease) into preoperative screening, rather than relying solely on standard guidelines or a single ultrasound view.

Comparing the two models’ performance, the model predicting the Perlas risk score outperformed the model predicting a full stomach from USG findings (AUC of 78.1% vs. 69.1%). One explanation is that Perlas scoring—by differentiating partial fluid or solid content—may capture more nuanced states of partial gastric filling, in contrast to a strict binary classification of “full” or “empty”. Indeed, the Youden’s index cutoffs of 0.13 and 0.18 are relatively low, which might mean the model flags risk at a lower predicted probability, aiming for higher sensitivity. Although neither model achieves perfect discrimination, the stronger AUC for the model predicting the Perlas risk score supports the idea that a multi-level assessment of gastric content can better distinguish intermediate risk states. However, because only nine patients had solid gastric content, we lacked the sample size needed to fully explore a three-category (Grade 0, 1, 2 or empty, fluid, solid) logistic model in both cases.

Our analysis revealed a Cohen’s Kappa value of 0.327, indicating fair agreement between the questionnaire-based predictions and USG findings. This implies that while the questionnaire can identify patients at risk for aspiration, its accuracy remains insufficient to replace USG in routine clinical practice. Nevertheless, the statistically significant Kappa value suggests that the questionnaire retains some predictive value and could serve as a quick screening tool in resource-limited settings—particularly in settings without ultrasound—alerting clinicians to potential risk factors such as early satiety or known cholelithiasis. This approach is in line with recent recommendations emphasizing individualized risk assessment when advanced diagnostic tools are not accessible [3,35]. When feasible, USG should remain the definitive method for identifying “full stomach” cases and guiding aspiration prevention strategies.

A key finding of this study is the short scanning time for USG. In physically fit patients with an empty stomach, the scanning could be completed in under one minute, totaling 2 min when accounting for patient interaction, abdominal exposure, and self-positioning on the right side. At the other end of the spectrum were elderly patients, who were slower to turn and required some comfort measures like support pads or heaters to ease or complete the full assessment. In a high-volume operating environment, a reliable imaging tool that can be performed swiftly offers a clear advantage. It should be mentioned that obesity did not interfere with the imaging but did necessitate an adjustment of depth focus. Also, waiting for a full peristaltic wave prolonged the imaging in some patients, regardless of their demographic characteristics or room temperature. Despite these occurrences, the median scanning time of 3 min remains short compared to the five minutes required for the questionnaire. This time difference may seem small but an extra one or two minutes per patient can quickly accumulate in large centers with hundreds of daily cases. Similarly, the questionnaire required less time in younger patients with no comorbidities or medications. In contrast, it took longer in elderly patients with multiple comorbidities and medications, where each medication had to be verified by checking the patient’s file, or electronic healthcare records.

A notable limitation is that the Perlas risk assessment requires imaging in both the supine and RLD positions [27,28]. In our cohort, 8.8% of patients could not tolerate RLD due to fractures, thereby rendering a full assessment impossible. This raises the possibility of missing clinically important “full stomach” cases in those who cannot be repositioned. While alternative scanning windows may be possible, a short term alternative may be minimal sedation or low-dose analgesics such as ketamine, fentanyl, or dexmedetomidine to facilitate patient comfort during repositioning. However, sedation itself may alter gastric motility or sphincter tonus if used at higher doses, and the feasibility of administering sedation preoperatively may vary due to patient characteristics such as obstructive sleep apnea syndrome or lung diseases. Future research could clarify whether such adaptations safely increase the percentage of patients eligible for comprehensive ultrasound assessment, without unnecessarily extending preparation times or introducing additional risks.

Several limitations must be acknowledged: First, we did not measure the gastric volume with orogastric tube aspiration, which could have provided a more definitive gold standard to validate the USG measurements. Second, we had only nine patients presenting with solid gastric content, preventing us from conducting a three-category analysis and forcing us to merge fluid and solid or no-risk and low-risk into a single category. Thus, while our results support the utility of volume formulas and the Perlas risk score, we were unable to fully assess whether more finely graded ultrasound findings—particularly for patients with solid content—might offer superior predictive value. Third, although 393 patients remained in our final regression models after excluding missing data, the remaining data are susceptible to subjectivity because of their reliance on patient-reported symptoms. Lastly, all USG measurements were performed by a single investigator, which reduces inter-operator variability but may limit generalizability to broader clinical settings.

## 5. Conclusions

USG is an efficient, reliable, and time-saving method for identifying residual gastric content, proving advantageous over traditional questionnaire-based methods. Despite its demonstrated advantages, two significant limitations must be noted. First, a subset of patients cannot undergo ultrasound assessment in the right lateral decubitus position, which is crucial for Perlas grading. Second, volume estimation formulas can yield implausible or negative values. Thus, clinicians should rely on direct USG visualization—potentially augmented by patient-reported risk factors such as early satiety—to enhance preoperative screening for aspiration risk. Future studies should prioritize refining gastric volume estimation and accommodate patients with positioning constraints.

## Figures and Tables

**Table 1:** Comparison of demographic and clinical characteristics between patients who could and could not tolerate the right lateral decubitus position.

Patient Characteristic	RLD Tolerant (n = 404)	RLD Not Tolerant (n = 45)	*p*-Value
Age, years	53 ± 15.7	54 ± 17.5	0.624
Sex (male), n (%)	258 (64%)	29 (64%)	1
BMI, kg/m^2^	29.7 ± 8.8	28.7 ± 6.3	0.437
ASA Physical Score			0.549
1	35 (8.7%)	2 (4.4%)	
2	238 (59%)	32 (71%)	
3	114 (28%)	10 (22%)	
4	17 (4.2%)	1 (2.2%)	
Fasting duration, hours	12.7 ± 3.9	12.5 ± 2.4	0.896
Time to surgery, days	7 (5–14)	10 (5–15)	0.386
Ward stay, n (%)	45 (11%)	4 (8.9%)	0.836
Previous GIS surgery, n (%)	64 (15.8%)	6 (13.3%)	0.823
History of cancer, n (%)	47 (11.6%)	4 (8.9%)	0.717
History of hypertension, n (%)	139 (34%)	17 (38%)	0.775
History of diabetes, n (%)	63 (16%)	4 (8.9%)	0.328
Early satiety, n (%)	12 (3%)	1 (2.2%)	1
Cholelithiasis, n (%)	17 (4.2%)	2 (4.4%)	1

ASA, American Society of Anesthesiologists; GIS, gastrointestinal system; BMI, body mass index.

**Table 2:** Comparison of patient characteristics based on ultrasound findings and Perlas risk grades.

	Group by USG Findings			Group by Perlas Risk Score			
Patient Characteristic	Empty (n = 338)	Non-Empty (n = 66)	*p*-Value	Grade 0 (n = 355)	Grade 1 (n = 37)	Grade 2 (n = 12)	*p*-Value
Age, years	53.1 ± 15.8	53.2 ± 14.8	0.922	53.1 ± 15.8	52.6 ± 13.7	52.3 ± 18.3	0.935
Sex (male), n (%)	212 (63%)	46 (70%)	0.348	220 (62%)	30 (81%)	8 (67%)	0.069
BMI, kg/m^2^	29.2 ± 6.3	30.9 ± 6.5	0.051 ^1^	29.5 ± 6.5	29.6 ± 4.9	30.3 ± 7.8	0.829
ASA physical score			0.188				0.209
1	32 (9.5%)	3 (4.5%)		32 (9%)	1 (2.7%)	2 (16.7%)	
2	200 (59.2%)	38 (57.6%)		207 (58.3%)	27 (73%)	4 (33.3%)	
3	90 (26.6%)	24 (36.4%)		100 (28.2%)	8 (21.6%)	6 (50%)	
4	16 (4.7%)	1 (1.5%)		16 (4.5%)	1 (2.7%)		
Fasting duration, hours	12.8 ± 4.1	11.9 ± 2.5	0.021 ^2^	12.8 ± 4.1	12 ± 2.5	11.4 ± 2.2	0.082
Time to surgery, days	7 (5–14)	10 (7–15)	0.270	7 (5–14)	7 (4–10)	12.5 (9.5–30) ^3^	0.037
Ward stay, n (%)	37 (11%)	8 (1.2%)	0.949	39 (10.9%)	4 (10.8%)	2 (16.6%)	0.826
Previous GIS surgery, n (%)	49 (14.5%)	15 (22.7%)	0.246	54 (15.2%)	8 (21.6%)	2 (16.6%)	0.595
History of cancer, n (%)	42 (12.4%)	5 (7.6%)	0.319	45 (12.6%)	1 (2.7%)	1 (8.3%)	0.167
Early satiety, n (%)							
Cholelithiasis, n (%)							

ASA, American Society of Anesthesiologists; GIS, gastrointestinal system; BMI, body mass index. ^1^ 95% CI, 0 to 3.5; ^2^ 95% CI, −0.13 to −1.64, corresponding to 6 min to 90 min; ^3^ significantly high compared to the other groups (*p* = 0.035).

**Table 3:** Summary of antrum area and estimated gastric volumes according to Perlas risk grades.

Patient Characteristic	Grade 1 (n = 37)	Grade 2 (n = 12)	*p*-Value
Antrum Area, cm^2^	16.3 (2.7–65.1)	26.8 (2.5–82.4)	0.103
Estimated gastric volume, ml			
Michiko formula	−297.9 (−625.5–110.7)	−251.1 (−578.7–157.5)	0.839
Bouvet formula	115.65 (15.34–208.34)	137.4 (23.16–213.28)	0.132
Perlas formula	211.9 (−11.52–937.78)	338.92 (17.42–1185.24)	0.072
Patients with negative estimated gastric volume			
Michiko formula	37 (100%)	20 (100%)	0.024
Bouvet formula	0 (0%)	0 (0%)	0.024
Perlas formula	3 (8.11%)	0 (0%)	0.492

**Table 4:** Logistic regression models to predict Perlas risk scores based on estimated gastric volumes.

Formula	Estimate	Std. Error	Probability	Df	AIC
Michiko formula	0.002431	0.001184	0.040	56	358.98
Bouvet formula	−0.009994	0.006847	0.144	56	75.552
Perlas formula	−0.002595	0.001273	0.041	56	73.096

Df = degrees of freedom, AIC = Akaike’s information criterion.

**Table 5:** Logistic regression summary for predicting non-empty stomach (USG findings), ordered by standardized error.

Formula	Estimate	Std. Error	Probability
(Intercept)	−0.17326	0.74296	0.815600
Early satiety	2.55389	0.73082	0.000475
Fasting duration	−0.11691	0.05815	0.044387
Female sex	−0.64804	0.33748	0.054828
Cholelithiasis	1.12826	0.61033	0.064514
Proteinuri	−1.94850	1.16539	0.094529
Previous GIS surgery	0.60464	0.36182	0.094697
Use of antacids	−0.95872	0.63018	0.128175
NIDDM	0.55670	0.38056	0.143510

Df = degrees of freedom, AIC = Akaike’s information criterion.

**Table 6:** Logistic regression summary for predicting Perlas risk score, ordered by standardized error.

Formula	Estimate	Std. Error	Probability
(Intercept)	−1.77350	1.37383	0.19673
Early satiety	2.72924	1.00043	0.00637
Female sex	−1.03290	0.47282	0.02892
Fasting duration	−0.10974	0.07359	0.13589
Use of antacids	−1.51995	1.03538	0.14210
Cancer surgery	1.40386	1.03696	0.17579

## Data Availability

The raw data supporting the conclusions of this article and the R source files used during the analysis will be made available by the authors on reasonable request.

## Abbreviations

The following abbreviations are used in this manuscript:

USG	Ultrasound
ROC	Receiver operating curve
AUC	Area under the curve
STROBE	Strengthening the reporting of observational studies in epidemiology
RLD	Right lateral decubitus

## Appendix A

### Appendix A.1

**Table A1:** English translation of the staff-administered questionnaire used for assessing patient risk factors and clinical data. This table is not intended for direct patient self-completion; items referencing ASA classification and specific drug categories require clarification by trained healthcare personnel. Healthcare staff should pose each question or consult the medical record, then record the findings accordingly.

Item	Prompt (Staff Use)	Staff Instructions	Responses/Notes
1. Cancer surgery?	“Is the scheduled procedure for cancer treatment?”	Check surgical notes or confirm with patient’s chart.	Yes/No
2. Previous surgeries	“Please list any previous surgeries or major procedures you have undergone.”	Reference the patient’s medical record; ask follow-up if unclear or incomplete.	Free text/Short answers
3. Ward or home?	“Where has the patient been monitored prior to surgery? Are they staying in the ward or at home?”	Determine current location.	Ward/Home/Other
4. Fasting period (hours)	“For how many hours has the patient been fasting before surgery (solids)?”	Inquire about the last solid meal time, cross-check with the patient or chart if needed.	Integer (hours)
5. ASA physical score	“What is the patient’s ASA classification (1–5, E)?”	Assigned by anesthesiologist or from the record.	ASA 1–5, E if applicable
6. Age	“What is the patient’s age in years?”	Record from the patient’s chart or ID, confirm if needed.	Integer (years)
7. Height	“Patient’s height (in cm or m).”	Measure or reference the official record.	Numeric (cm/m)
8. Weight	“Patient’s weight (in kg).”	Weigh the patient or confirm from chart if recent measurement is available.	Numeric (kg)
9. Gender	“Patient’s gender.”	Confirm with medical record or by direct query if necessary.	Male/Female/Other
10. Body type	“Based on appearance and records, is the patient cachectic, normal, or obese?”	Assess visually, check BMI if needed.	Cachectic/Normal/Obese
11. Smoking history	a. Duration, b. Amount, c. Has the patient stopped smoking?, d. Smoked during fasting?	(a) Ask how many years the patient has smoked(b) Packs/day or similar(c) Confirm if/when they quit(d) If they smoked after starting to fast, note the approximate time.	a) Free text (years)b) Free text (packs/day)c) Yes/Nod) Yes/No
12. Diabetes (DM) history	a. Type of diabetes (Type 1, Type 2, etc.), b. Duration (years), c. Treatment (diet, OAD, insulin), d. Latest HbA1c (%), e. Preoperative blood glucose, f. Maximum BG at home, g. Minimum BG at home	Review medical record or ask the patient if they are aware; confirm with lab results if available.	Record each item as numeric or short text.
13. Retinopathy	“Does the patient have diabetic retinopathy or similar microvascular eye disease?”	Check the ophthalmology record or previous diagnoses.	Yes/No/Not applicable
14. Dysphagia	“Has the patient reported difficulty swallowing?”	Directly ask the patient or check GI consult notes.	Yes/No
15. Reduced appetite (Dysappetite)	“Does the patient have a poor appetite or reduced interest in eating?”	Confirm by patient interview or nutritional consult.	Yes/No
16. Early satiety	“Does the patient feel full after only a small amount of food?”	Ask patient or check GI consult.	Yes/No
17. Upper abdominal distention	“Does the patient experience bloating or distention in the upper abdomen after small meals?”	Ask the patient or see if GI notes mention this.	Yes/No
18. Persistent nausea/vomiting	“Does the patient frequently experience ongoing nausea or vomiting episodes?”	Clarify how often and if it’s new or chronic.	Yes/No
19. Postural hypotension	“Does the patient report dizziness or lightheadedness upon standing up from sitting or lying down?”	Interview the patient; confirm if there’s an official diagnosis or mention in records.	Yes/No
20. Numbness in hands/feet	“Does the patient have numbness, tingling, or decreased sensation in the extremities?”	Inquire or check neurology consult if relevant.	Yes/No
21. Charcot arthropathy	“Has the patient been diagnosed with Charcot arthropathy (neuropathic joint disease)?”	Check orthopedic or diabetes records.	Yes/No/Unsure
22. Excessive sweating	“Does the patient experience excessive or profuse sweating without obvious triggers?”	Ask if the patient finds themselves sweating abnormally (e.g., at rest).	Yes/No
23. Reduced sweating (anhidrosis)	“Does the patient rarely or never sweat, even in hot conditions?”	Ask patient or note any dermatology consult.	Yes/No
24. Drug history	Has the patient used any of the following:–Steroids–Antiemetics–Antihypertensives–Antacids–Antidepressants–Antipsychotics–Other?	Cross-check with pharmacy record or medication list.	Check all that apply/add free text as needed
25. Comorbidities	Check or list all that apply:Hemorrhagic/Ischemic stroke, Hemiplegia, Hypo-/Hyperthyroidism, COPD, Asthma, Seasonal allergic rhinitis, Hypertension, CAD, PADIBS, Crohn, UC, Constipation, Diarrhea, Cholelithiasis, Chronic pancreatitis, CKD, Proteinuria, Renal replacement therapy	Confirm from the patient’s medical records. For uncertain items (e.g., constipation/diarrhea), confirm with patient interview.	Select all that apply/short text for additional notes
26. Self-care	(a) Dementia?(b) Can the patient handle self-care independently?(c) Can the patient feed themselves?(d) Anxiety?	Evaluate cognition or rely on caretaker or patient statement.	(a) Yes/No(b) Yes/No(c) Yes/No(d) Yes/No
27. Poor nutritional condition	“Does the patient appear cachectic, have thin subcutaneous tissue, or a scaphoid abdomen?”	Check nutritional assessment, if available.	Yes/No
28. Premedications	“Were any of these administered before surgery?–Antibiotics–Antacids–Prokinetics–Anxiolytics”	Ask or check the pre-op medication chart.	Check all that apply

### Appendix A.2

**Table A2:** Summary of questionnaire responses.

Item	Total (n = 404)	Empty (n = 338)	Non-Empty (n = 66)
Surgery Type			
Urologic Surgery	105 (26%)	87	18
Ureteroscopy (URS)	39	31	8
Transurethral Resection of Bladder Tumor	15	14	1
Transurethral Resection of Prostate	13	8	5
Cystoscopy	9	9	-
Urethral Stricture Repair	5	5	-
Percutaneous Nephrolithotomy	5	3	2
Radical Prostatectomy	5	5	-
Double-J Stent Removal	3	2	1
Nephrectomy	3	2	-
Double-J Stent Insertion	2	3	-
Cystectomy	2	2	-
Bladder Botox Injection	1	1	-
Orchiectomy	1	1	-
Retrograde Intrarenal Surgery	1	-	1
Ureteral Tumor Resection	1	1	-
Vertebral Surgery	95 (23.5%)	82	12
Lumbar Disc Surgery	70	62	8
Spinal Stabilization	12	8	4
Cervical Disc Herniation Surgery	9	9	-
Lumbar Stenosis Surgery	2	1	-
Lumbar Abscess Drainage	1	1	-
Spondylodiscitis Surgery	1	1	-
Abdominal Surgery	60 (14.9%)	46	14
Cholecystectomy	29	25	4
Inguinal Hernia Repair	20	15	5
Endoscopic Retrograde Cholangiopancreatography	5	3	2
Sleeve Gastrectomy	2	0	2
Epigastric Hernia Repair	1	1	-
Colon Cancer Surgery	1	1	-
Colorectal Fistula Repair	1	1	-
Total Abdominal Hysterectomy	1	1	-
General Surgery	59 (14.6%)	49	10
Thyroidectomy	33	29	4
Breast Lump Excision	6	4	2
Parathyroidectomy	6	5	1
Axillary Lymph Node Dissection	5	2	1
Umbilical Hernia Repair	4	4	1
Hydrocele Repair	2	2	-
Inguinal Lymph Node Dissection	2	1	-
Lipoma Excision	1	-	2
Cancer Surgery	25 (6.2%)	15	3
Mastectomy	11	2	2
Laryngectomy	4	2	1
Dissection of Lymph Nodes	3	3	-
Rectal Cancer Surgery	2	2	-
Cranial Tumor Surgery	1	1	-
Nasal Tumor Surgery	1	1	-
Paraganglioma Surgery	1	1	-
Mandibular Tumor Surgery	1	1	-
Metastasectomy	1	1	-
Lower Extremity	18 (4.45%)	1	-
Metatarsal Fracture Surgery	4		
Femur Fracture Repair	3	10	1
Tibia Fracture Repair	3	2	-
Material Removal Surgery	2	1	-
Fibula Fracture Repair	1	3	1
Wrist Fracture Surgery	1	2	1
Wrist Graft Placement	1	1	-
Knee Prosthesis Surgery	1	-	1
Hip Prosthesis Surgery	1	1	-
Knee Arthroscopy	1	1	-
Coronary Artery Bypass Surgery	15 (3.7%)	14	1
Thoracic Surgery	10 (2.45%)		
Video-Assisted Thoracoscopic Surgery	7	61	1
Pneumonectomy	1	1	-
Lobectomy	1	1	-
Mediastinoscopy	1	-	1
Anal Surgery	8 (2%)	8	-
Anal Fissure Surgery	2	2	-
Pilonidal Sinus Surgery	2	2	-
Anal Fistula Surgery	1	1	-
Anal Sphincter Relaxation Surgery	1	1	-
Hemorrhoid Surgery	1	1	-
Rectovaginal Fistula Repair	1	1	-
Upper Extremity	7 (1.7%)	5	2
Radius Fracture Repair	3	2	1
Humerus Fracture Repair	2	2	-
Ulnar Fracture Repair	1	-	1
Wrist Laceration Repair	1	1	-
Otorhinolaryngology Surgery	2 (0.5%)		
Functional Endoscopic Sinus Surgery	1	1	-
Voice Prosthesis Replacement	1	1	-
History of cancer	47 (12.4%)	42 (12.8%)	5 (7.6%)
Time to surgery, days	7 (5–14)	7 (5–14)	10 (7–15)
Previous GIS surgery	64 (15.8%)	49 (14.5%)	15 (22.7%)
Ward or home			
Home	359 (88.9%)	301 (89.1%)	58 (87.9%)
Ward	45 (11.1%)	37 (10.9%)	8 (12.1%)
Fasting period, hours	12.7 ± 3.9	12.8 ± 4.1	11.9 ± 2.5
ASA physical score			
1	35 (8.7%)	32 (9.5%)	3 (4.5%)
2	238 (58.9%)	200 (59.2%)	38 (57.6%)
3	114 (28.2%)	90 (26.6%)	24 (7.1%)
4	17 (4.2%)	16 (4.7%)	1 (1.5%)
Age	53.1 ± 15.7	53.1 ± 15.8	53.3 ± 14.9
Height	167.6 ± 12.8	167.8 ± 15.8	166.6 ± 15.6
Weight	82.8 ± 15.8	82.1 ± 15.8	86.2 ± 15.6
Gender			
Male	258 (63.9%)	212 (62.7%)	46 (69.7%)
Female	146 (36.1%)	126 (37.3%)	20 (30.3%)
Body type			
Normal	230 (56.9%)	196 (58%)	34 (51.5%)
Obese	122 (30.2%)	94 (27.8%)	28 (42.4%)
Cachectic	52 (12.9%)	48 (14.2%)	4 (6.1%)
Active smoker	101 (25%)	86 (25.4%)	15 (22.7%)
Duration	20 (10–30)	20 (10–30)	20 (10–30)
Amount, packs/day	0 (0–0)	0 (0–0)	0 (0–1)
Previous smoker	64 (15.8)	51 (15.1)	13 (19.7%)
Quitted smoking when	10 (1.75–20)	10 (1.5–20)	9 (4–15)
Smoked during fasting period	66 (16.3)	51 (15.1%)	15 (22.7%)
Diagnosis of Dabetes mellitus	63 (15.3%)	49 (14.5%)	14 (21.2%)
Type of Diabetes			
Non-insulin-dependent diabetes	63 (15.3%)	49 (14.5%)	14 (21.2%)
Duration of diabetes	7.5 (2–10)	6 (2–10)	9 (3.25–13.75)
Latest HbA1c, %	7.5 (6.5–9.6)	7 (6.5–9.3)	8.2 (7.48–9.8)
Diabetes treatment type			
Diet only	1 (0.2%)	1 (0.3%)	–
Oral antidiabetic only	44 (10.9%)	34 (10.1%)	10 (15.2%)
Oral antidiabetic ± insulin	14 (3.5%)	12 (3.6%)	2 (3%)
Insulin only	4 (1%)	3 (0.9%)	1 (1.5%)
Daily insulin dose	32 (28–42)	32 (30–43)	24 (22–25)
Preoperative blood glucose, mg/dL	140 (120–160)	140 (120–220)	150 (124–178)
Maximum blood glucose at home, mg/dL	200 (170–260)	200 (155–265)	225 (180–258)
Minimum blood glucose at home, mg/dL	100 (90–120)	100 (90–120)	120 (100–150)
Retinopathy	11 (2.7%)	10 (3%)	1 (1.5%)
Dysphagia	1 (0.2%)	-	1 (1.5%)
Reduced appetite	10 (2.5%)	7 (2.1%)	3 (4.5%)
Early satiety	12 (3%)	4 (1.2%)	8 (12.1%)
Upper abdominal distention	30 (7.4%)	22 (6.5%)	8 (12%)
Persistent nausea/vomiting	10 (2.5%)	8 (2.4%)	2 (3%)
Postural hypotension	10 (2.5%)	9 (2.7%)	1 (1.5%)
Numbness in hands/feet	21 (5.2%)	16 (4.7%)	5 (7.6%)
Charcot arthropathy	1 (0.2%)	1 (0.3%)	-
Excessive sweating	-	-	-
Reduced sweating	-	-	-
Medications			
Corticosteroids	4 (1%)	4 (1.2%)	-
Antiemetics	-	-	-
Antihypertensives	149 (36.9%)	123 (36.4%)	26 (39.4%)
Antacids	40 (9.9%)	37 (10.9%)	3 (4.5%)
Antidepressants	16 (4%)	14 (4.1%)	2 (3%)
Antipsychotics	3 (0.7%)	3 (0.9%)	-
Other medications			
Acetylsalicylic acid	47 (11.6%)	37 (10.9%)	10 (15.2%)
Levothyroxine	30 (7.4%)	25 (7.4%)	5 (7.6%)
Statins	9 (2.2%)	8 (2.4%)	1 (1.5%)
Clopidogrel	10 (2.5%)	6 (1.8%)	4 (6%)
Warfarin	5 (1.25%)	4 (1.2%)	1 (1.5%)
Ipratropium bromide	10 (2.5%)	9 (2.7%)	1 (1.5%)
Betahistine	4 (1%)	3 (0.9%)	1 (1.5%)
Hemorrhagic stroke	-	-	-
Ischemic stroke	2 (0.5%)	2 (0.6%)	-
Hemiplegia	-	-	-
Hypothyroidism	30 (7.4%)	25 (7.4%)	5 (7.6%)
Hyperthyroidism	8 (2%)	6 (1.8%)	2 (3%)
Chronic Obstructive Pulmonary Disease	10 (2.5%)	9 (2.7%)	1 (1.5%)
Asthma	12 (3%)	11 (3.3%)	1 (1.5%)
Seasonal allergic rhinitis	-	-	-
Hypertension	139 (34.4%)	114 (33.7%)	25 (37.9%)
Coronary artery disease	44 (10.9%)	34 (10.1%)	10 (15.2%)
Peripheral artery disease	-	-	-
Inflammatory bowel disease	1 (0.2%)	1 (0.3%)	-
Crohn’s disease	-	-	-
Ulcerative colitis	-	-	-
Constipation	55 (13.6%)	42 (12.4%)	13 (19.7%)
Diarrhea	5 (1.2%)	5 (1.5%)	-
Cholelithiasis	17 (4.2%)	12 (3.6%)	5 (7.6%)
Chronic Kidney Disease	15 (3.7%)	14 (4.1%)	1 (1.5%)
Renal replacement therapy	-	-	-
Dementia	1 (0.2%)	-	1 (1.5%)
Can handle self-care independently	3 (0.7%)	2 (0.6%)	1 (1.5%)
Can feed themselves	1 (0.2%)	1 (0.3%)	-
Anxiety	3 (0.7%)	3 (0.9%)	-
Poor nutritional condition	7 (1.7%)	5 (1.5%)	2 (3%)
Premedications administered before surgery			
Antibiotics			
Cefazolin	4	4	-
Ceftriaxon	2	1	1
Metronidazole	1	1	1
Moxifloxacin	1	-	1
Ciprofloxacin	1	-	1
Meropenem	1	-	1
Antacids			
Pantoprazol	11	9	2
Ranitidin	2	1	1
Prokinetics	-	-	-
Anxiolytics	-	-	-
Tramadol	3	2	1
Enoxaparine	4	3	1
